# Comparison of Heart and Lung Doses According to Tumor Bed Boost Techniques in Early-Stage Left-Sided Breast Cancer: Simultaneous Integrated Boost versus Sequential Boost

**DOI:** 10.3390/medicina58070873

**Published:** 2022-06-29

**Authors:** Myungsoo Kim, Nam Kwon Lee, Suk Lee, Jinho Hwang

**Affiliations:** 1 Department of Radiation Oncology, Incheon St. Mary’s Hospital, College of Medicine, The Catholic University of Korea, Seoul 06591, Korea; mskim0710@gmail.com (M.K.); b75365817@gmail.com (J.H.); 2 Department of Radiation Oncology, Korea University Anam Hospital, Korea University College of Medicine, Seoul 02841, Korea; sukmp@korea.ac.kr

**Keywords:** breast cancer, radiotherapy, volumetric modulated arc therapy, simultaneous integrated boost

## Abstract

*Background and Objectives*: The boost dose to the tumor bed after whole breast irradiation (WBI) can be divided into sequential boost (SEQ) and simultaneous integrated boost (SIB). SIB using modern radiation therapy (RT) techniques, such as volumetric modulated arc therapy, allow the delivery of a highly conformal dose to the target volume and has a salient ability to spare at-risk organs. This study aimed to compare the radiation dose delivered to the heart and lungs according to boost technique and tumor bed location. *Materials and Methods*: RT planning data of 20 patients with early-stage left-sided breast cancer were used in this study. All patients were treated with volumetric modulated arc therapy after breast-conserving surgery with a sentinel lymph node biopsy. For each patient, two different plans, whole breast irradiation with simultaneous integrated boost (WBI-SIB) and sequential boost after WBI (WBI-SEQ), were generated. To compare the dose received by each organ at risk (OAR), dose-volume histogram data were analyzed. The mean dose (D_mean_) and volume of each organ that received x Gy (Vx) were calculated and compared. *Results*: For the heart, the V10 was lower for the WBI-SIB plan than for the WBI-SEQ plan (5.223 ± 1.947% vs. 6.409 ± 2.545%, *p* = 0.008). For the left lung, the V5 was lower in the WBI-SIB plan than for the WBI-SEQ plan (27.385 ± 3.871% vs. 32.092 ± 3.545%, *p* < 0.001). The D_mean_ for the heart and left lung was lower for the WBI-SIB plan than for the WBI-SEQ plan (heart: 339.745 ± 46.889 cGy vs. 413.030 ± 52.456 cGy, *p* < 0.001; left lung: 550.445 ± 65.094 cGy vs. 602.270 ± 55.775 cGy, *p* < 0.001). *Conclusions*: The WBI-SIB plan delivered lower radiation doses to the heart and left lung than the WBI-SEQ plan in terms of D_mean_ and low-dose volume in hypofractionated RT of early-stage left-sided breast cancer patients. Furthermore, a large radiation dose per day may be advantageous, considering the radiobiologic aspects of breast cancer. Long-term follow-up data are needed to determine whether the dosimetric advantages of the WBI-SIB plan can lead to clinically improved patient outcomes and reduced late side effects.

## 1. Introduction

Whole breast irradiation (WBI) following breast-conserving surgery (BCS) is the current standard treatment for early-stage breast cancer, and a patient’s risk for recurrence is evaluated to determine whether to administer a boost dose to the tumor bed [1]. Tumor bed boost timing can be divided into sequential boost (SEQ), which is applied after WBI (WBI-SEQ), and simultaneous integrated boost (SIB), which is applied simultaneously (WBI-SIB).

Strict dose constraints are required in modern radiation therapy (RT) planning [2,3,4] due to the association between radiation dose to the heart and late heart disease. The heart is anatomically located closer to the target volume in cases of left-sided breast cancer than in cases of right-sided breast cancer, which requires strategies to minimize the radiation dose delivered to the heart. Modern RT techniques, such as volumetric modulated arc therapy (VMAT), allow the delivery of a highly conformal dose to the target volume and have a salient ability to spare organs at risk (OAR) compared to two-dimensional RT or three-dimensional conformal RT techniques [5]. The advantages of SIB in RT planning for breast cancer have already been demonstrated in several studies [6,7,8,9]; however, to date, few studies conducted have dosimetrically compared SEQ and SIB [10,11,12].

We compared doses delivered to the heart and lungs via the two boost techniques and analyzed which boost techniques were superior according to the target volume location of the tumor bed boost (inner versus outer quadrant) in terms of the dose delivered to the heart and lungs.

## 2. Materials and Methods

### 2.1. Patients 

This single-center study was performed at Incheon St. Mary’s Hospital. RT planning data of 20 patients with early-stage left-sided breast cancer were used in this study. All patients underwent VMAT between January 2021 and March 2022 after BCS with a sentinel lymph node biopsy. The eligibility criteria for the study included pT1-2 and N0 disease. Age at diagnosis and histologic subtype were not restricted. Patients treated with any neoadjuvant chemotherapy before BCS and those with a previous history of breast augmentation were excluded. This study was approved by the Institutional Review Board of the Catholic University of Korea at Incheon St. Mary’s Hospital (approval number: OC22EIDI0043), which waived the requirement for informed consent owing to the study’s retrospective nature.

### 2.2. Treatment Planning and Target Volume Delineation

**Figure 1:**
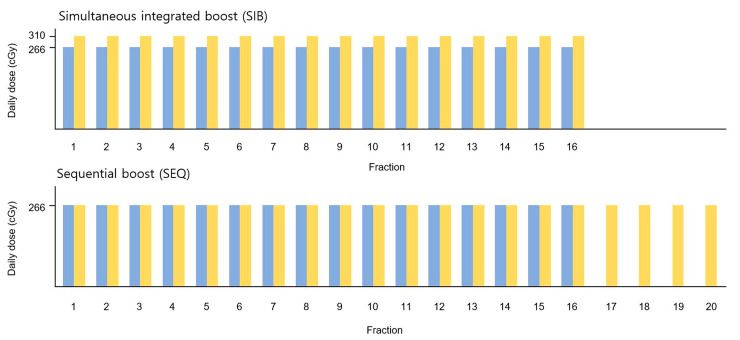
Schematic diagram of the treatment plans. The prescription doses for PTV_WB_ and PTV_Boost_ are shown in blue and yellow, respectively. PTV, planning target volume; WB, whole breast.

Hypofractionated (HF) RT was planned using the Eclipse^TM^ treatment planning system and was administered using Halcyon^TM^ (Varian Medical Systems, Palo Alto, CA, USA), which offers a 6 MV flattening-filter-free photon beam. For WBI, the clinical target volume (CTV) and planning target volume (PTV_WB_) were delineated by a single radiation oncologist according to the European Society for Radiotherapy and Oncology consensus guidelines [13]. PTV_WB_ was cropped to 3 mm from the body contour. For the tumor bed boost, surgical clips were delineated using computed tomography to define the lumpectomy cavity, and CTV_Boost_ was defined by adding 1 cm around the clips and seroma. PTV_Boost_ was defined by adding a 0.5 cm margin to CTV_Boost_. Two different plans, WBI-SIB and WBI-SEQ, were generated for each patient. The prescription doses and biologically effective doses (BED_3_) for each plan are summarized in Table 1. A schematic of each treatment plan is shown in Figure 1.

The prescription dose was administered at the isodose line to encompass at least 95% of the PTV, while limiting maximum doses to less than 107%. Two partial arcs with single-isocenter VMAT plans were generated for the WBI. When performing SEQ, the collimator angle was adjusted according to the PTV_Boost_ location. The VMAT plans were optimized using the Eclipse photon optimization algorithm, and Acuros XB (version 16.1) was used for dose calculation. Dose constraints were prescribed based on institutional guidelines (Table 2).

### 2.3. Assessment of Plan Quality

Three plan quality metrics proposed by the Radiation Therapy Oncology Group (RTOG) were used in this study: homogeneity index (HI), conformity index (CI), and quality of coverage (QOC) (Table 3) [14,15]. HI measures the uniformity of dose distribution in the target volume; an HI ≤ 2 indicates that the plan does not deviate from the protocol. CI explains how well the dose distribution conforms to the shape of the target volume and has an ideal value of 1. If CI > 1, then the irradiated volume is greater than TV, i.e., overtreatment; if CI < 1, then the irradiated volume is smaller than TV, i.e., undertreatment. A QOC > 0.9 indicates that over 90% of the isodose covers the target volume and the plan does not deviate from the protocol [16]. To compare the doses delivered to the OARs, dose-volume histogram data were analyzed. The mean doses (D_mean_) and volume of each organ that received x Gy (Vx) were calculated and compared.

### 2.4. Statistical Analysis

Wilcoxon signed-rank tests were used to compare non-parametric data. IBM SPSS Statistics for Windows (version 27.0; IBM Corp., Armonk, NY, USA) was used for the statistical analyses, and all statistical tests were considered statistically significant at *p* values < 0.05.

## 3. Results

**Figure 2:**
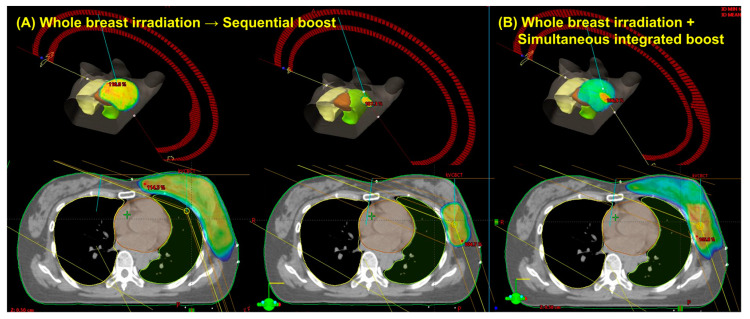
Dose distribution and three-dimensional view of a representative case using the sequential boost technique (**A**) versus the simultaneous integrated boost technique (**B**).

Patient and tumor characteristics are summarized in Table 4. In total, 20 women had pathologically confirmed early-stage left-sided breast cancer. All patients presented with clinically node-negative disease and had negative sentinel lymph node biopsies. Among the 20 patients, 14 (70.0%) received hormone suppression therapy. The mean PTV_WB_ and PTV_boost_ were 664.8 cm^3^ (range, 196.3–1158.5 cm^3^) and 70.5 cm^3^ (range, 33.8–142.2 cm^3^), respectively. The dose distribution and three-dimensional view of a typical patient receiving the WBI-SEQ and WBI-SIB plans are shown in Figure 2.

### 3.1. Assessment of Plan Quality

Plan quality was evaluated based on the criteria described in Table 3. The optimization algorithm was balanced for PTV coverage and the values for the WBI-SIB and WBI-SEQ plans were found to be acceptable. The HI values for the WBI-SIB and WBI-SEQ plans were 1.323 ± 0.027 and 1.446 ± 0.020, respectively. According to the RTOG protocol, both plans were considered to comply with the protocol. CI values for the WBI-SIB and WBI-SEQ plans were 0.958 ± 0.158 and 0.980 ± 0.008, respectively. Plans with a CI of 1 was the ideal value, and values between 0.9 and 1.0 were considered within minor deviation, but acceptable. The QOC values for the WBI-SIB and WBI-SEQ plans were 0.986 ± 0.015 and 1.001 ± 0.115, respectively; both plans complied with the protocol. Our institutional guidelines for dose constraints were satisfied for all patients.

### 3.2. WBI-SIB versus WBI-SEQ Plan

**Figure 3:**
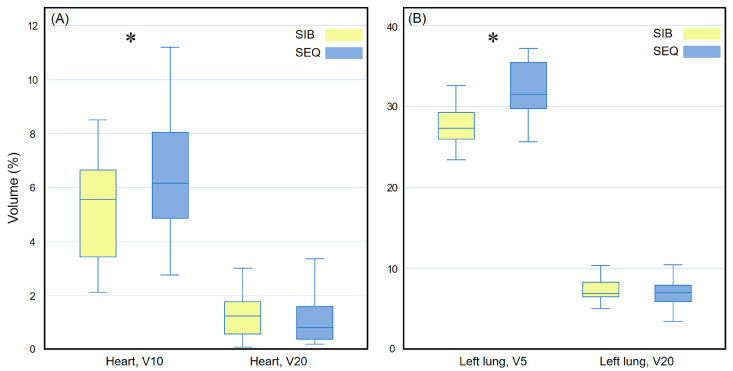
Box plots comparing the volume indices for (**A**) the heart and (**B**) the left lung between the WBI-SIB and WBI-SEQ plans. Statistical analyses were performed using the Wilcoxon signed-rank test. Asterisks (*) indicate statistical significance at *p* values < 0.05. SEQ, sequential boost; SIB, simultaneous integrated boost; WBI, whole breast irradiation.

Figure 3 compares the heart and left lung doses received via the WBI-SIB and WBI-SEQ plans. For the heart, the V10 was lower for the WBI-SIB plan than for the WBI-SEQ plan (5.223 ± 1.947% vs. 6.409 ± 2.545%, *p* = 0.008), whereas the V20 values did not differ significantly between the WBI-SIB and WBI-SEQ plans (1.191 ± 0.672% vs. 1.057 ± 0.848%, *p* = 0.161). As the dose increased, the difference in the irradiated volumes between the two treatment plans remained small. For the ipsilateral lung, the V5 was lower for the WBI-SIB plan than for the WBI-SEQ plan (27.385 ± 3.871% vs. 32.092 ± 3.545%, *p* < 0.001). The V20 values did not differ significantly between the WBI-SIB and WBI-SEQ plans (7.277 ± 1.898% vs. 7.124 ± 2.027%, *p* = 0.588).

**Figure 4:**
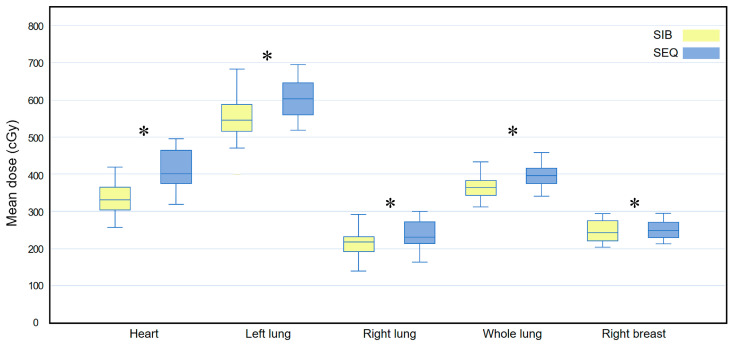
Box plots comparing the mean dose (D_mean_) delivered to all at-risk organs between the WBI-SIB and WBI-SEQ plans. Statistical analyses were performed using the Wilcoxon signed-rank test. Asterisks (*) indicate statistical significance at *p* values < 0.05.SEQ, sequential boost; SIB, simultaneous integrated boost; WBI, whole breast irradiation.

Figure 4 compares the D_mean_ for all OARs between the WBI-SIB and WBI-SEQ plans. The D_mean_ for all OARs was lower for the WBI-SIB plan than for the WBI-SEQ plan (heart: 339.745 ± 46.889 cGy vs. 413.030 ± 52.456 cGy, *p* < 0.001; left lung: 550.445 ± 65.094 cGy vs. 602.270 ± 55.775 cGy, *p* < 0.001; right lung: 217.640 ± 39.688 cGy vs. 239.340 ± 36.679 cGy, *p* = 0.001; whole lung: 363.470 ± 39.930 cGy vs. 398.310 ± 32.527 cGy, *p* < 0.001; and right breast: 245.625 ± 29.065 cGy vs. 255.970 ± 34.177 cGy, *p* = 0.025).

### 3.3. Subgroup Analysis: Inner Quadrant

For the heart dose, the WBI-SIB plan was slightly but non-significantly better than the WBI-SEQ plan for V10 (6.309 ± 1.391 vs. 7.314 ± 2.849, *p* = 0.074); for V20, there was no statistically significant difference between the WBI-SIB and WBI-SEQ plans (1.508 ± 0.715 vs. 1.428 ± 0.921, *p* = 0.575). For the left lung dose, the WBI-SIB plan was better than the WBI-SEQ plan for V5 (26.611 ± 3.550 vs. 31.139 ± 3.814, *p* = 0.007); for V20, there was no statistically significant difference between the WBI-SIB and WBI-SEQ plans (6.671 ± 1.946 vs. 6.170 ± 1.989, *p* = 0.153).

**Figure 5:**
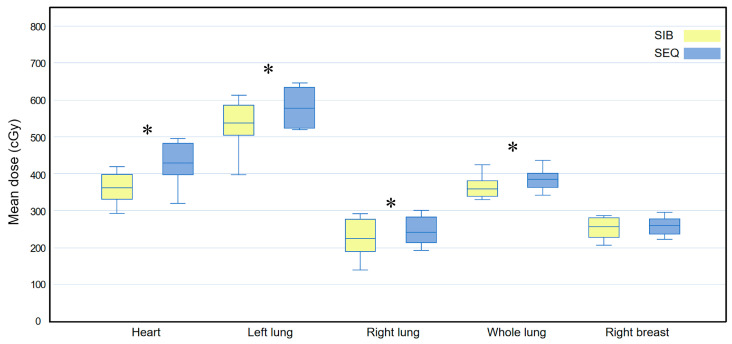
Box plots comparing the mean dose (D_mean_) delivered to all organs at risk between the WBI-SIB and WBI-SEQ plans in a subgroup analysis of the inner quadrant. Statistical analyses were performed using the Wilcoxon signed-rank test. Asterisks (*) indicate statistical significance at *p* values < 0.05. SEQ, sequential boost; SIB, simultaneous integrated boost; WBI, whole breast irradiation.

Figure 5 compares the D_mean_ for all OAR between the WBI-SIB and WBI-SEQ plans. The D_mean_ values for the heart, left lung, right lung, and whole lung were lower for the WBI-SIB plan than for the WBI-SEQ plan (heart: 360.930 ± 39.652 cGy vs. 429.770 ± 58.904 cGy, *p* = 0.005; left lung: 533.330 ± 64.527 cGy vs. 575.800 ± 52.888 cGy, *p* = 0.009; right lung: 227.410 ± 51.038 cGy vs. 245.330 ± 37.562 cGy, *p* = 0.037; and whole lung: 357.770 ± 47.530 cGy vs. 385.970 ± 29.697 cGy, *p* = 0.005). For the right breast, the D_mean_ did not differ between the WBI-SIB and WBI-SEQ plans (253.130 ± 28.958 cGy vs. 267.510 ± 40.350 cGy, *p* = 0.114). 

### 3.4. Subgroup Analysis: Outer Quadrant

In terms of heart dose, the WBI-SIB plan was superior to the WBI-SEQ plan for V10 (4.137 ± 1.855 vs. 8.503 ± 1.930, *p* = 0.017); in contrast, for V20, there was no statistically significant difference between the two plans (0.873 ± 0.707 vs. 0.685 ± 0.601, *p* = 0.092). For the left lung dose, the WBI-SIB plan was superior to the WBI-SEQ plan for V5 (28.159 ± 4.207 vs. 33.046 ± 3.156, *p* = 0.009), while for V20, there was no statistically significant difference between the two plans (7.882 ± 1.732 vs. 8.078 ± 1.642, *p* = 0.445).

**Figure 6:**
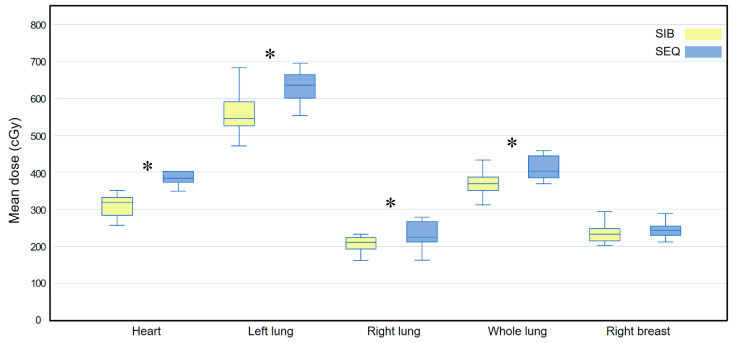
Box plots comparing the mean dose (D_mean_) delivered to all organs at risk between the WBI-SIB and WBI-SEQ plans in a subgroup analysis of the outer quadrant. Statistical analyses were performed using the Wilcoxon signed-rank test. Asterisks (*) indicate statistical significance at *p* values < 0.05. SEQ, sequential boost; SIB, simultaneous integrated boost; WBI, whole breast irradiation.

Figure 6 compares the D_mean_ for all OARs between the two plans. The D_mean_ values for the heart, left lung, right lung, and whole lung were lower for the WBI-SIB plan than for the WBI-SEQ plan (heart: 318.560 ± 45.516 cGy vs. 396.290 ± 41.350 cGy, *p* = 0.005; left lung: 567.560 ± 64.268 cGy vs. 628.740 ± 47.044 cGy, *p* = 0.007; right lung: 207.870 ± 64.268 cGy vs. 233.350 ± 36.736 cGy, *p* = 0.007; and whole lung: 369.170 ± 32.163 cGy vs. 410.650 ± 31.830 cGy, *p* = 0.005, respectively). For the right breast, the D_mean_ did not differ between the WBI-SIB and WBI-SEQ plans (238.120 ± 28.629 cGy vs. 244.430 ± 23.277 cGy, *p* = 0.114).

## 4. Discussion

In this study, we focused on the importance of heart and lung radiation doses in the treatment of early-stage left-sided breast cancer and analyzed how boost techniques and tumor bed location affected RT planning and its dosimetric effects on the heart and left lung. Our results showed that the WBI-SIB plan resulted in a lower D_mean_ for all OAR than the WBI-SEQ plan and particularly reduced the low-dose volume of the heart and left lung.

Although RT is an integral part of breast cancer treatment, long-term attention to the risk of late complications, such as heart disease, is required, particularly for left-sided breast cancer patients [2,3,4,17]. Carlson et al. [17] reported the results of a WELCARE (Women’s Environmental Cancer and Radiation Epidemiology) follow-up study of self-reported incident cardiovascular disease in women with left-sided breast cancer. The 27.5-year cumulative incidence of coronary artery disease was 2.5-fold higher in women with left-sided breast cancer than in those with right-sided breast cancer (95% confidence interval, 1.3–4.7). Darby et al. conducted another large population-based case-control study of major coronary events, such as myocardial infarction, coronary revascularization, or death of ischemic heart disease [2], and reported that the major coronary events rate increased by 7.4% per Gy with increasing D_mean_ to the heart. Several studies using modern RT techniques such as VMAT have been conducted in an effort to reduce the dose delivered to the heart [18,19].

SIB allows the simultaneous delivery of a differential dose per fraction to different target volumes and offers several advantages such as a higher biologically effective dose to PTV_TB_, shorter overall treatment time than SEQ, and highly homogeneous and conformal dose distributions compared with SEQ or field-in-field techniques [20,21]. Several recent studies have examined intensity-modulated radiation therapy (IMRT) with SIB plans and have shown dosimetric advantages [5,7]. Guerrero et al. [6]. compared the conventional treatment of WBI (45 Gy in 25 fractions) plus SEQ (20 Gy in 10 fractions) and a biologically equivalent alternative plan of WBI (45 Gy in 25 fractions) with SIB (60 Gy in 25 fractions) using IMRT and reported that the latter provided good coverage of the target volume and reduced the volume of excessively high doses to the breast, especially for patients with deep-seated tumors.

Based on four published randomized trials [22,23,24,25,26,27], the National Comprehensive Cancer Network guidelines recommend a HF dose of 40–42.5 Gy in 15–16 fractions for the WBI [1]. Few reports are available on the dosimetric feasibility of HF-SIB, which has not yet been widely adopted clinically for breast cancer. Yu T et al. [28] dosimetrically compared HF-WBI with SEQ versus SIB in the supine and prone positions using three-dimensional conformal RT with the field-in-field technique. The dose prescribed for WBI was 40.05 Gy in 15 fractions, while that of the tumor bed was 9.6 Gy in 3 fractions for SEQ and 48 Gy in 15 fractions for SIB. Regardless of the position, SIB-HF-WBI resulted in better target coverage and a lower dose to the OARs. Breast cancer is estimated to have a low α/β ratio, similar to that of late-reacting normal tissue [22,23,24]. The low estimated α/β ratio for breast cancer indicates that it is probably more sensitive to the effect of fraction size than most other tumors; therefore, hypofractionation for breast cancer may have a therapeutic advantage over conventional fractionation.

The heart is normally located behind and slightly to the left of the breastbone, and the left anterior descending coronary artery (LAD) runs along its surface. When the PTV_TB_ is in the inner quadrant, the distance between the heart or LAD and the PTV_TB_ is anatomically closer than that in the outer quadrant. Bouchardy et al. [29] compared breast cancer-specific and cardiovascular mortality between the inner and outer quadrants using data of 1245 women in the population-based Geneva Cancer Registry. In their study, patients with inner quadrant breast cancer had a 2.5-fold higher risk for cardiovascular mortality than those with outer quadrant breast cancer (95% confidence interval, 1.1–5.4). Since the heart dose can increase as the tumor bed becomes closer to the heart, we assessed which treatment plan has the potential benefit of either WBI-SIB or WBI-SEQ according to tumor bed location. It was not possible to determine which treatment plan was better for the heart dose depending on the tumor bed location, and it was confirmed that the WBI-SIB plan had potential advantages over the WBI-SEQ plan in the low-dose region, regardless of the tumor bed location.

This study had a number of limitations. We agree that the small sample size weakened the statistical power and the lack of the evaluation of acute and late complications limited this study, which is an inherent limitation of RT planning studies. Through a further randomized controlled study, we expect to compare late complications and cosmetic outcomes of the WBI-SIB versus WBI-SEQ plans.

## 5. Conclusions

In this study, we have confirmed that the WBI-SIB plan offered lower doses to OARs in terms of D_mean_ and low-dose volume in HF RT of patients with left breast cancer. WBI-SIB offers a shortened overall treatment period and increases patient convenience. Furthermore, a large radiation dose per day may be advantageous, considering the radiobiologic aspects of breast cancer. Long-term follow-up data are needed to determine whether these dosimetric advantages of the WBI-SIB plan can lead to clinically improved patient outcomes and reduced late side effects.

## Figures and Tables

**Table 1:** Biologically effective dose with an assumed value of α/β = 3 Gy.

	Prescription	BED_3_ (Gy)
Simultaneous integrated boost (SIB) SIB-PTV_WB_ SIB-PTV_Boost_	42.56 Gy in 16 fractions 49.60 Gy in 16 fractions	80.30 100.85
Sequential boost (SEQ) SEQ-PTV_WB_ SEQ-PTV_Boost_	42.56 Gy in 16 fractions 10.64 Gy in 4 fractions	80.30 100.37

BED, biologically effective dose; PTV = planning target volume; WB, whole breast.

**Table 2:** Dose constraints for organ at risk.

Organ at Risk	Dose Constraints
Heart	V30 < 5%
	D_mean_ < 5 Gy
Ipsilateral lung	V20 < 20%
	D_mean_ < 7 Gy
Contralateral lung	D_mean_ < 3 Gy
Whole lungs	V20 < 10%
	D_mean_ < 5 Gy
Contralateral breast	D_mean_ < 3 Gy

D_mean_, mean dose; Vx, volume receives x Gy.

**Table 3:** Volume-based indices for plan quality assessment.

Index for PTV	Formula	Reference Value
Homogeneity index	I_max_/RI	≤2: per protocol >2.0 and ≤2.5: minor deviation >2.5: major deviation
Conformity index	V_RI_/TV	1: ideal value 1.0–2.0: per protocol 0.9–1.0 or 2.0–2.5: minor deviation <0.9 or >2.5: major deviation
Quality of coverage	I_min_/RI	1: ideal value ≥0.9 and <1.0: per protocol ≥0.8 and <0.9: minor deviation <0.8: major deviation

I_max_, maximum isodose in the target; I_min_: minimum isodose in the target; PTV, planning target volume; RI, reference isodose; TV, target volume; V_RI_, reference isodose volume.

**Table 4:** Patient and tumor characteristics (*n* = 20).

Characteristic	Classification	Number of Patients (%)
Age (years)	Median (range)	54 (39–79) *
Histology	Invasive ductal carcinoma Metaplastic carcinoma	19 (95.0) 1 (5.0)
Histologic grade	I II III	4 (20.0) 9 (45.0) 7 (35.0)
Tumor location	Left inner quadrant Left outer quadrant	10 (50.0) 10 (50.0)
T classification	T1_mi_ T1 T2	2 (10.0) 11 (55.0) 7 (35.0)
Hormone receptor status	ER- or PR-positive ER- and PR-negative	14 (70.0) 6 (30.0)
HER2/neu ^†^ receptor	Positive Negative	0 (00.0) 20 (100.0)

* Values are shown as numbers (percentage) unless otherwise noted. † Immunohistochemistry 3+ or fluorescent in situ hybridization positive. ER, estrogen receptor; HER2, human epidermal growth factor receptor 2; PR, progesterone receptor.

## Data Availability

Data are available upon reasonable request.
